# Correlation of Aquaporins and Transmembrane Solute Transporters Revealed by Genome-Wide Analysis in Developing Maize Leaf

**DOI:** 10.1155/2012/546930

**Published:** 2012-09-26

**Authors:** Xun Yue, XiangYu Zhao, YuKui Fei, Xiansheng Zhang

**Affiliations:** ^1^College of Information Sciences and Engineering, Shandong Agricultural University, Shandong, Taian 271018, China; ^2^State Key Laboratory of Crop Biology, College of Life Sciences, Shandong Agricultural University, Shandong, Taian 271018, China

## Abstract

Aquaporins are multifunctional membrane channels that facilitate the transmembrane transport of water and solutes. When transmembrane mineral nutrient transporters exhibit the same expression patterns as aquaporins under diverse temporal and physiological conditions, there is a greater probability that they interact. In this study, genome-wide temporal profiling of transcripts analysis and coexpression network-based approaches are used to examine the significant specificity correlation of aquaporins and transmembrane solute transporters in developing maize leaf. The results indicate that specific maize aquaporins are related to specific transmembrane solute transporters. The analysis demonstrates a systems-level correlation between aquaporins, nutrient transporters, and the homeostasis of mineral nutrients in developing maize leaf. Our results provide a resource for further studies into the physiological function of these aquaporins.

## 1. Introduction

Water can take different paths on its way through the leaf, in addition to radial water flux, water movement across leaf cell membranes is important for water homeostasis, increasing cell volume, maintaining turgor during expansion, regulating the opening and closure of stomata, and controlling leaf movement [1]. Water movement through cell membranes is facilitated by water channels called aquaporins. Plant aquaporins exhibit multiplicity and diversity, and fall into seven subfamilies loosely based on intracellular locations and sequence similarities: the plasma membrane intrinsic proteins (PIPs), tonoplast intrinsic proteins (TIPs), NOD26-like intrinsic proteins (NIPs), small, basic intrinsic proteins (SIPs), the GlpF-like intrinsic proteins (GIPs), hybrid intrinsic proteins (HIP), and the uncategorized X intrinsic proteins (XIP) [2].

Plant aquaporins are significant not only in plant-water relations, but also in physiological aspects such as nutrient transport and metal/metalloid toxicity [3, 4]. Flexas et al. provided evidence for the *in vivo* involvement of *NtAQP1* in mesophyll CO_2_ conductance, suggesting a significant role for PIPs in CO_2_ diffusivity [5]. Ludewig and Dynowski have shown that AtTIP1;1 and *AtTIP1;2* conduct H_2_O_2_ when heterologously expressed in yeast [6]. Azad et al. described *TgTIP1;1*- and *TgTIP1;2*-mediated H_2_O_2_ conductance by fluorescence assay in *Tulipa gesneriana*. Recent studies have investigated the selectivity mechanisms of aquaporins, nutrient transporters and homeostasis of mineral nutrients in most plant groups [7]. Hove and Bhave performed a comprehensive analysis of all plant aquaporins, which have been proven to transport ammonia, boron, carbon dioxide, hydrogen peroxide, silicon, and urea [8]. However, their studies focused on aquaporin structures at atomic resolution; there exists little information about the development and growth-dependent expression of aquaporins in leaves and how this relates to nutrient transporters and the homeostasis of mineral nutrients [9].

The use of genomics has resulted in many insights into the fully differentiated state of the maize (*Zea mays*) leaf [10–12]. Breeze et al. used microarray analysis to obtain a high-resolution time-course profile of gene expression during the development of a single leaf over a 3-week period to senescence [13]. Recently, RNA-Seq has emerged as a powerful new technology for transcriptome analysis [14]. RNA-Seq is particularly advantageous for low-abundance transcripts, for which microarrays are generally insensitive [15]. Matthieu et al. presented a detailed and quantitative analysis of expression in leaf zones, via functional characterization and determination of tissue-specific expression, for a selected group of candidate barley (*Hordeum vulgare*) aquaporins. These analyses enabled allocation of particular roles during leaf development to specific aquaporin isoforms and proposal of factors that influence their expression [16]. By Illumina sequencing of the maize leaf transcriptome [17], more than 120 million reads were mapped to define gene structure and alternative splicing events, and to quantify transcript abundance, along a leaf developmental gradient. Differential mRNA processing events for most maize genes were detected.

In this study, we use genome-wide analysis and coexpression network-based approaches to examine the wide range of selectivity profiles of aquaporins and associated transmembrane solute transporters in developing maize leaf. We identify specific maize aquaporins related to specific transmembrane solute transporters. Our results provide a resource for further studies into the physiological function of these aquaporins.

## 2. Materials and Methods

### 2.1. Materials and Data

Aimed at examining the transcriptional network associated with the development of maize leaf, Pinghua et al. defined gene structure, alternative splicing events, and quantified transcript abundance along a leaf developmental gradient [17]. We used their methods as a template for gene expression analysis. Tissue from leaf three at nine days after planting, three hours into the L period, was collected from four segments: basal (1 cm above the leaf three ligule), transitional (1 cm below the leaf two ligule), maturing (4 cm above the leaf two ligule), and mature (1 cm below the leaf three tip). All data were sequenced and analyzed using an Illumina Genome Analyser 2. RNA-Seq data have been deposited in the NCBI short read archive under accession number SRA012297.

### 2.2. Method and Computational Pipeline for Genome-Wide Analysis

**Figure 1:**
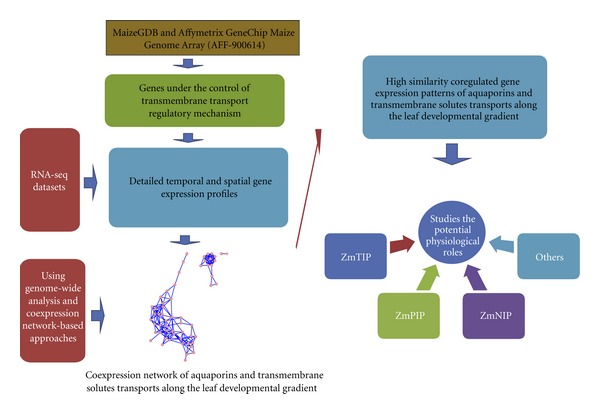
Schematic of computational pipeline for genome-wide analysis and coexpression network-based approaches.

In this study, our research concentrates on mining the systems-level correlation of aquaporins and associated transmembrane nutrient solute transporters along a leaf developmental gradient. We know that aquaporins and transmembrane mineral nutrient transporters are all under the control of transmembrane transport regulatory mechanisms (GO:0055085). Thus, when transmembrane mineral transporters exhibit the same expression patterns as aquaporins under four segments: basal, transitional, maturing, and mature, there is a greater probability that they interact [18–22]. Using genome-wide analysis and coexpression network-based approaches, systems-level correlation of maize aquaporins, nutrient transporters, and homeostasis of mineral nutrients can be examined. The computational pipeline for genome-wide analysis is shown in Figure 1.

First, we identified characteristic maize aquaporin family and transmembrane transporters under the control of transmembrane transport regulatory mechanisms (GO:0055085) from the MaizeGDB and Affymetrix GeneChip Maize Genome Array (AFF-900614). Next, detailed temporal and spatial gene expression profiles for aquaporins and nutrient transporters along the developing maize leaf were obtained though RNA-Seq data. Then, high similarity coregulated gene expression patterns were studied and mined by coexpression network-based approaches. Finally, the AmiGO Gene Ontology Database and iHOP (Information  Hyperlinked  over Proteins) were examined to characterize correlation of *ZmTIPs, ZmPIPs, and ZmNIPs* with associated transmembrane solute transport.

### 2.3. Rank-Based Network Construction Method

A range of statistical and computational methodologies have been used to extract novel meaning from large biological datasets. Common approaches include both differential expression across conditions and the calculation of correlations between gene expression levels across a large number of samples [18, 23]. The coexpression network-based approach, termed coexpression analysis, considers all samples together and establishes connections between genes based on all available information. Based on the methods from Ruan et al. [24], our computational methodology uses a simple but robust rank-based network construction method. In this experiment, the leaf transcriptome mRNA was isolated from each of the four developmental zones, basal (1 cm above the leaf three ligule), transitional, (1 cm below the leaf two ligule), maturing (4 cm above the leaf two ligule), and mature (1 cm below the leaf three tip). Each gene is associated with a set of expression values, called gene expression profile. We first calculate the Pearson's correlation coefficient between every pair of genes. We then define a correlation-based similarity score to measure the average coexpression between a gene and other members. For each gene, we rank all other genes by their similarity. We calculate the scores for all the genes. The gene rank of each gene is the position of the score after sorting the list of scores in descending order. We then select these genes to the genes that are most similar to it. For two nodes to be considered as coexpressed, their expression profiles need to satisfy the following (1) Pearson's correlation coefficient is higher than min_cc (2) One gene is within the max_rank most correlated gene of the other. Cytoscape is used for network visualization and analysis [25]. After we generated the coexpression network, the statistics of the networks (such as, average path lengths, diameters, clustering coefficients, node degree distributions) should be analyzed. But, in this study, rank-based network construction method are used mainly to demonstrate a systems-level correlation between aquaporins, nutrient transporters, and the homeostasis of mineral nutrients in developing maize leaf. So, the topological properties of coexpression network will be provided for further studies.

On the other hand, the method has two parameters to be defined in order to run the algorithm: (1) min_cc: threshold on Pearson's correlation coefficient, (2) max_rank: threshold on the ranks of the correlation coefficient values. In particular, genes in one functional pathway may be strongly mutually coexpressed, while genes in another functional pathway may be only weakly coexpressed. After some preliminary tests with the algorithm, we choose a stringent threshold on Pearson's correlation coefficient (min_cc = 0.95), it means we attempt to connect the strongly mutually coexpressed genes in one functional pathway, we choose a larger threshold on the ranks of the correlation coeffient values (max_rank = 50), it means we attempt to choose the max subset of the most correlated genes. This rank-based network construction method has successfully elucidated gene function in *Arabidopsis thaliana* from genome-wide coexpression networks, leading to the identification of genes essential in the life cycle of Arabidopsis [21] and regulation of seed germination [20]. 

## 3. Results 

### 3.1. Identification of Candidate Aquaporins and Transmembrane Solute Transporters in Maize

Genome projects have identified 33 aquaporins in maize: 13 PIPs split into 6 PIP1s and 7 PIP2s, 11 TIPs, 6 NIPs, and 3 SIPs. No GIPs, HIPs, or XIPs have been found in maize (see supplemental file 2 in Supplementary Material available online at doi:10.1155/2012/546930). Existing maize aquaporin families were retrieved from the MaizeGDB website. Phylogenetic analysis of maize aquaporins, together with sequences from Arabidopsis and rice, has been performed by Katsuhara et al. [26]. In addition to the aquaporin families, 812 maize transmembrane transport genes (GO:0055085) were identified from the MaizeGDB and Affymetrix GeneChip Maize Genome Array (see supplemental file 1). 

In the RNA-Seq dataset, which quantified transcript abundance along a leaf developmental gradient, 24 mRNA maize isoforms were detected, representing three maize aquaporin subfamilies: 11 plasma membrane (*PIP*), 8 tonoplast (*TIP*), and 5 NOD26-like (*NIP*). The others, *ZmPIP1;2*, *ZmPIP2;7*, *ZmNIP5;1*, *ZmTIP1;1*, *ZmTIP4;1*, *ZmTIP4;3*, *ZmSIP1;1*, *ZmSIP1;2*, and *ZmSIP2;1*, showed hardly any or no expression. Surprisingly, *ZmPIP1;3* and *ZmPIP1;4* were not discernible. Of the maize transmembrane transporters (GO:0055085), only 481 were examined for channel activity by heterologous expression along the leaf developmental gradient (see supplemental file 3).

### 3.2. Expression Patterns of Maize Aquaporins along a Leaf Developmental Gradient

As shown in Table 1, the expression profiles of aquaporins along a leaf developmental gradient were mostly statistically significant, and expression differed by orders of magnitude.

After examining the dynamic expression patterns of aquaporins in four maize leaf developmental zones, we were interested in studying the specifically expressed genes in each leaf developmental zone. A gene that was expressed at low levels or not detected in the stage was defined as a specifically expressed gene in this stage. Eight genes, *ZmTIP4;3*(GRMZM2G146627), *ZmTIP4;1*(GRMZM2G103945), *ZmTIP1;1*(AAC09245), *ZmSIP1;1*(GRMZM2G113470), *ZmSIP1;2*(GRMZM2G060922), *ZmSIP2;1*(GRMZM2G175038), *ZmPIP2;7*(AAK26763, zma:542645), *ZmNIP5-1*(GRMZM2G000471), showed hardly any or no expression in four developmental maize leaf zones. *ZmTIP4;4*, *ZmTIP3;2*, *ZmNIP1;1*, and *ZmNIP3;1* were specifically expressed in the mature leaf zone, while *ZmNIP2;1* and *ZmTIP4;2* were specifically expressed in the leaf basal.

**Figure 2:**
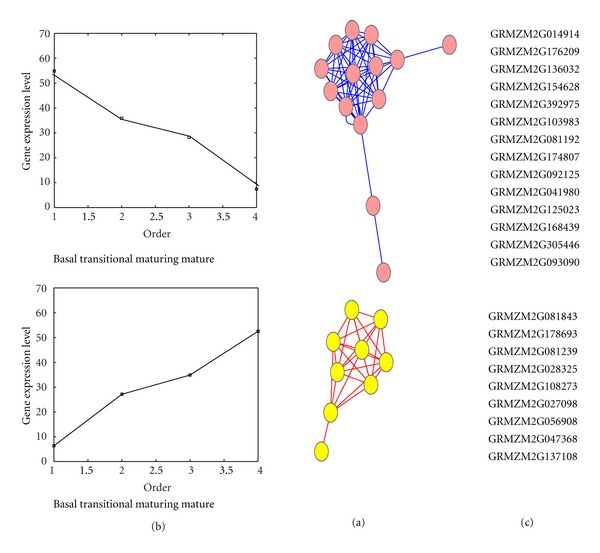
Expression patterns of maize aquaporins along a leaf developmental gradient. (a) The transcriptional network of maize aquaporin families identified in leaf developmental gradient. (b) Two expression profiles of aquaporins along a leaf developmental gradient, one modules showed the highest expression in the basal and transitional zone showed much lower or hardly detectable expression in the maturing and mature zone. another modules showed the highest expression in maturing and mature leaf tissue, and (c) genes in two modules.

Based on the gene expression profiles of the 24 maize aquaporins along a leaf developmental gradient, a coexpression network-based approach was used to construct coexpression networks. Cytoscape was used to create Figure 2. This approach partitioned the network into two modules, indicating a strong modular structure. We then examined the temporal gene expression profiles of the two modules. As shown in Figure 2, the modules identified by our method contained significant transcripts that showed the most dramatic changes. One modules includes *ZmPIP2;1*(GRMZM2G014914), *ZmNIP3;1*(GRMZM2G176209), *ZmPIP1;6*(GRMZM2G136032), *ZmPIP2;4*(GRMZM2G154628), *ZmPIP1;3/ZmPIP1;4*(GRMZM2G392975), *ZmTIP3;2*(GRMZM2G103983), *ZmPIP2;3*(GRMZM2G081192), *ZmPIP1;1*(GRMZM2G174807), *ZmPIP2;2*(GRMZM2G092125), *ZmNIP1;1*(GRMZM2G041980), *ZmTIP2;3*(GRMZM2G125023), *ZmTIP1;2*(GRMZM2G168439), *ZmTIP3;1*(GRMZM2G305446), *ZmTIP4;4*(GRMZM2G093090), 14 genes which showed the highest expression in the basal and transitional zone showed much lower or hardly detectable expression in the maturing and mature zone. While the opposite applied to another modules which are *ZmPIP1;5 *(GRMZM2G081843), *ZmPIP2;5*(GRMZM2G178693), *ZmNIP2;3*(GRMZM2G081239), *ZmNIP2;1*(GRMZM2G028325), *ZmTIP4;2*(GRMZM2G108273), *ZmTIP2;1*(GRMZM2G027098), *ZmTIP2;2*(GRMZM2G056908), *ZmPIP2;6*(GRMZM2G047368), *ZmNIP2;2*(GRMZM2G137108), those genes which showed the highest expression in maturing and mature leaf tissue. These results do not seem surprising considering that there are two groups of aquaporins, one positively and the other negatively regulated during different stages of leaf development.

By comparing the expression profiles of ZmNIPs (*ZmNIP1;1, ZmNIP2;1, ZmNIP2;2, ZmNIP2;3, ZmNIP3;1*) in four maize leaf developmental zones (basal, transitional, maturing, mature), it was evident that the ZmNIP family members are expressed in distinct patterns during leaf development (Table 1). We noticed that *ZmNIP2;1, ZmNIP2;2,* and *ZmNIP2;3* were expressed at low levels at the base of the leaf and gradually increased to reach their highest levels at the leaf tip. In contrast, *ZmNIP1;1* and *ZmNIP3;1* were expressed at their highest levels at the base of the leaf and decreased significantly to low levels at the leaf tip. The most notable gene, which was upregulated during the whole course of the experiment, was *ZmNIP2;2.*

Interestingly, with the exception of *ZmPIP2;7*, which was not expressed, *ZmPIP1*s and *ZmPIP2*s showed the highest expression in the four leaf base zones. However, these genes showed significant changes in expression levels in the four stages. *ZmPIP1;1*, *ZmPIP1;3*, *ZmPIP1;4*, *ZmPIP2;1*, *ZmPIP2;2*, *ZmPIP2;3*, and *ZmPIP2;4* showed their highest expression in the base, with much lower expression in the mature zone. The opposite applied to *ZmPIP1;5*, *ZmPIP1;6*, *ZmPIP2;5*, and *ZmPIP2;6*, which showed their highest expression in the mature region. Expression in the transitional and maturing zones was at an intermediate level. *ZmPIP1;5* showed the most dramatic change in relative expression (a more than 30-fold increase).

Compared with the expression profiles of the *ZmNIP*s and *ZmPIP*s, with the exception of *ZmTIP1;1*, which was not expressed, the *ZmTIP*s showed distinct patterns during leaf development. We noticed that *ZmTIP3;1* showed very low expression or expression near the limit of detection along the leaf developmental gradient. *ZmTIP2;3* and *ZmTIP4;4* were expressed at low levels in the mature leaf zone, and *ZmTIP4;2* showed expression near the limit of detection at the leaf base. *ZmTIP1;2*, *ZmTIP2;1*, *ZmTIP2;2,* and *ZmTIP3;2* showed the most dramatic changes in relative expression (more than 10-fold increases).

### 3.3. *ZmTIP*s and Associated Transmembrane Solute Transport

**Figure 3:**
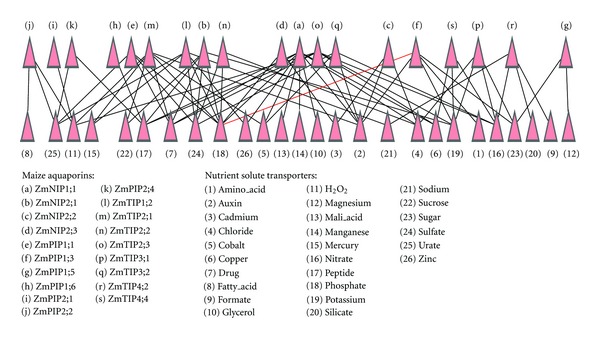
Correlation of maize aquaporins and associated nutrient solute transporters in developing maize leaf. The detailed analysis can be found in Sections 3.3, 3.4, and 3.5.

While TIPs are predominantly located in the tonoplast, some ZmTIPs are found in specialized organelles such as protein storage vacuoles, lytic vacuoles and small vacuoles, [27]. As shown in Figure 3, we observed significant relationships between *ZmTIP* genes and neutral solute transporters that exhibited the same expression patterns in the developing leaf. These included nitrate, hydrogen peroxide (H_2_O_2_), sulfate, auxin, sucrose, mercury, drug, sugar, chloride, peptide and oligopeptide transmembrane transporters, and metal ion transporters such as iron, potassium, copper, and cobalt.

**Nitrate**

Chopin et al. showed that the ATNRT2.7 nitrate transporter protein is localized to the vacuolar membrane and plays a specific role in nitrate accumulation in the seed [28]. Our results showed that *NRT2.5* (nitrate transporter 2.5, GRMZM2G455124) exhibited the same expression patterns as *ZmTIP2;3*, *ZmTIP3;2,* and *ZmTIP4;4*.

**Hydrogen Peroxide (H_2_O_2_)**

Evidence for the H_2_O_2_ permeability of aquaporins comes from the work of [29] and [7]. Dynowski et al. have shown that the plant TIPs AtTIP1;1 and AtTIP1;2 conduct H_2_O_2_ when heterologously expressed in yeast [29]. Azad et al. described TgTIP1;1- and TgTIP1;2-mediated H_2_O_2_ conductance by fluorescence assay in *Tulipa gesneriana* [7]. Our results showed that the C2C2(Zn)-GATA transcription factor family (AC202864.3_FG002, EntrezGene:100279625) exhibited the same expression patterns as *ZmTIP1;2*, and the bZIP transcription factor family (GRMZM2G445575, EntrezGene:100192007) exhibited the same expression patterns as *ZmTIP2;1*. These may be involved in sequence-specific DNA-binding transcription factor activity (GO:0003700) that catalyzes the reaction: vanillyl alcohol + O_2_ = vanillin + hydrogen peroxide in developing leaves. Our results also showed that mitochondrial SBP40 (GRMZM2G102314) exhibited the same expression patterns as *ZmTIP2;3*. This protein may be involved in single-stranded DNA binding (GO:0003697) that catalyzes the reaction: methanol + O_2_ = formaldehyde + H_2_O_2_ in developing leaves.

**Sulfate**

Transmembrane sulfate transport is performed by a family of high-affinity sulfate transporters that includes *SULTR1;1, SULTR1;3, SULTR1;2, SULTR3;1, SULTR3;2, SULTR3;4, SULTR3;5, SULTR4;1,* and *SULTR4;2*. Kataoka et al. described how SULTR3;5 facilitates the root-to-shoot transport of sulfate through the vasculature [30]. Yoshimoto et al. provided evidence that the Sultr1;3 transporter plays an important role in the loading of sulfate into the sieve tube, initiating the source-to-sink translocation of sulfur nutrients in Arabidopsis [31]. Our results showed that *SULTR2;1* (sulfate transporter2;1, GRMZM2G042171) exhibited the same expression patterns as *ZmTIP2;2*, and *SULTR3;4* (sulfate transporter 3;4, GRMZM2G444801) exhibited the same expression patterns as *ZmTIP3;2*. These genes may be involved in transmembrane sulfate transport in the developing leaf.

**Auxin**

ABCB19 of *A. thaliana* (ATPase multidrug resistance protein) belongs to the multidrug resistance-like (MDR) or B group of the ATP-binding cassette (ABC) transporter superfamily, and mediates polar auxin transport in stems and roots. Lewis et al. suggested that cotyledon expansion during the establishment of photoautotrophic growth depends on ABCB19-mediated auxin import [32]. Our results showed that *ABCB19* (GRMZM2G085236) exhibited the same expression patterns as *ZmTIP2;3* and *ZmTIP3;2*, and *ABCB1* (GRMZM2G315375, EntrezGene:1003840) exhibited the same expression patterns as *ZmTIP2;3*. These ABC transporters may be involved in multidrug transport and resistance systems in the developing leaf. We also observed a significant relationship between *ZmTIP2;1* and an auxin efflux carrier family protein (GRMZM2G112598).

**Sucrose**

*SUT4* (sucrose transporter 4, GRMZM2G307561, EntrezGene:100240688) exhibited the same expression patterns as *ZmTIP2;1* and may be involved in sucrose transmembrane transporter activity (GO:0008515) in developing leaves.

**Mercury**

Vacuolar membranes from different species have been commonly characterized by a high, mercury-sensitive osmotic water permeability [33]. TIPs, which function as mercury-sensitive channels and can account for up to 40% of total intrinsic TP protein content [34], supposedly play an important role in this vacuolar function. Our results showed that a heavy-metal-associated domain-containing protein (GRMZM2G096008) exhibited the same expression patterns as *ZmTIP2;1* and may be involved in mercury ion transmembrane transporter activity (GO:0015097) in the developing leaf.

We also observed significant relationships between *ZmTIP2;1* and a MATE efflux family protein (GRMZM2G170128), and *ZmTIP2;3* and another MATE efflux family protein (GRMZM2G423884). These may be involved in transmembrane drug transport (GO:0015238). *ATPLT5* (polyol transporter 5, GRMZM2G481021) exhibited the same expression patterns as *ZmTIP3;2* and may be involved in sugar and substrate-specific transmembrane transporter activity (GO:0022891). GPT2 glucose-6-phosphate transmembrane transporter (GRMZM2G009223, EntrezGene:100281048) exhibited the same expression patterns as *ZmTIP2;1* and *ZmTIP2;2*. Lipoprotein (AC234165.1_FG002, EntrezGene:100280698) exhibited the same expression patterns as *ZmTIP2;2*. Uncharacterized GPI-anchored protein (GRMZM2G041645) exhibited the same expression patterns as *ZmTIP2;3* and may be involved in voltage-gated chloride channel activity (GO:0005247) in developing leaves. APP (poly adp-ribose polymerase, GRMZM2G099231;) exhibited the same expression patterns as *ZmTIP2;3* and may be involved in nucleic acid binding (GO:0003676) that catalyzes the reaction: xanthine + NAD^+^ + H_2_O = urate + NADH + H^+^ in the developing leaf. Peptide transporter PTR2-B, a proton-dependent oligopeptide transport (POT) family protein (GRMZM2G316889, EntrezGene:100381733) exhibited the same expression patterns as *ZmTIP2;3* and may be involved in oligopeptide transport (GO:0006857) during leaf development.

**Iron**

Photosynthesis, heme biosynthesis, and Fe-S cluster assembly all take place in the chloroplast, and all require iron. Reduction of iron via a membrane-bound Fe(III) chelate reductase is required before it is transported across membranes. Jeong and Cohu reported that the Arabidopsis ferric reductase oxidase (FRO) family, FRO7, localizes to the chloroplast. Their results provided molecular evidence that FRO7 plays a role in chloroplast iron acquisition and is required for efficient photosynthesis in young seedlings and survival under iron-limiting conditions [35]. Wu et al. showed that the six *AtFRO*s encode iron chelate reductases that function in iron homeostasis in Arabidopsis. *AtFRO2* displayed the highest iron reduction activity among the *AtFRO*s investigated; further demonstrating that *AtFRO2* is a major iron reductase gene in Arabidopsis. *AtFRO2* and *AtFRO3* were mainly expressed in the roots, *AtFRO5* and *AtFRO6* in shoots and flowers, *AtFRO7* in cotyledons and trichomes, and the transcription of *AtFRO8* was specific to leaf veins [36]. Our results showed that ferric reductase-like transmembrane component, *FRO7* (GRMZM2G068557, EntrezGene:100281526;) exhibited the same expression patterns as *ZmTIP4;2*.

Other significant relationships we observed are as follows: a flavin-containing monooxygenase family protein/FMO family protein (GRMZM2G423886, EntrezGene:100272315), a sarcosine oxidase family protein (GRMZM2G428628), and an oxidoreductase family protein (GRMZM2G174773) exhibited the same expression patterns as *ZmTIP4;4* and *ZmTIP2;3* and may be involved in potassium ion transport (GO:0006813). *sks17* (SKU5 Similar 17, GRMZM2G043301) exhibited the same expression patterns as *ZmTIP2;3* and *ZmTIP4;4* and may be involved in copper ion binding (GO:0005507). *BAG6* (bcl-2-associated athanogene 6, GRMZM2G063162) exhibited the same expression patterns as *ZmTIP2;1* and may be involved in cellular cobalt ion homeostasis (GO:0006877) in the developing leaf.

### 3.4. *ZmPIP*s and Associated Transmembrane Solute Transport

PIPs are localized to the plasma membrane and facilitate the movement of water into and out of cells. Flexas et al. provided evidence for the *in vivo* involvement of NtAQP1 in mesophyll CO_2_ conductance, suggesting a significant role for PIPs in CO_2_ diffusivity [5]. Fitzpatrick and Reid established that at least 50% of boron uptake could be facilitated by two barley aquaglyceroporins, HvPIP1;3, and HvPIP1;4 [37]. In fact, most PIP2s show substantial water channel activity, whereas PIP1s either facilitate the movement of neutral solutes or increase water channel activity when coexpressed with PIP2s [3]. We observed significant relationships between *ZmPIP* genes and the transport of transmembrane solutes, including urate, hydrogen peroxide (H_2_O_2_), phosphate, sulfate, sugars, peptides, fatty acids, amino acids, and the metal ions potassium, magnesium, sodium, zinc, and mercury. These results are shown in Figure 3.

**Urate**

Gaspar et al. showed that ZmPIP1-5b has a low aquaporin activity when expressed in Xenopus oocytes. However, a special feature of ZmPIP1-5, when compared with other plant PIPs, is its capacity to transport urea [38]. Interestingly, our results show that glycine-rich RNA-binding protein 2 (GRMZM2G080603, EntrezGene:542725) exhibited the same expression patterns as *ZmPIP1;1* and may be involved in catalysis of the reaction: xanthine + NAD^+^ + H_2_O = urate + NADH + H^+^. These showed their highest expression in the four leaf base zones. Our results also showed that ATP-dependent RNA helicase SUV3 (GRMZM2G078275) and helicase domain-containing protein (GRMZM2G373175) exhibited the same expression patterns as *ZmPIP2;4*. These may also be involved in catalysis of the reaction: xanthine + NAD^+^ + H_2_O = urate + NADH + H^+^ in developing leaves. In addition, *LOS4* (low expression of osmotically responsive genes 4, GRMZM2G000823) exhibited the same expression patterns as *ZmPIP1;1* and *ZmPIP2;1*, and RNA recognition motif (RRM)-containing protein (GRMZM2G071589, EntrezGene:100383130) exhibited the same expression patterns as *ZmPIP2;2*.

**Hydrogen Peroxide (H_2_O_2_)**

TSA: *Zea mays* contig14775 mRNA sequence (UniGene:Zm.86314) and TSA: *Zea mays* contig01669 mRNA sequence (UniGene:Zm.42122) exhibited the same expression patterns as *ZmPIP2;2*. These may be involved in catalysis of the reaction: vanillyl alcohol + O_2_ = vanillin + hydrogen peroxide in the developing leaf.

**Phosphate**

Phosphate transporter (GRMZM2G015401) exhibited the same expression patterns as *ZmPIP1;1*, MAG1 (MAIGO 1, GRMZM2G109315, EntrezGene:100192764) exhibited the same expression patterns as *ZmPIP2;1*, and GRMZM2G152827 (EntrezGene:541617) exhibited the same expression patterns as *ZmPIP2;1*. These genes may be involved in mitochondrial phosphate transport in developing leaves.

**Sulfate**

In contrast to *ZmNIP*s, our results show that *SULTR3;4* (sulfate transporter 3;4, GRMZM2G444801, EntrezGene:100281787) exhibited the same expression patterns as *ZmPIP1;1* and *ZmPIP2;1* and may be involved in sulfate transmembrane transport in the developing leaf.

**Sugar**

STP (sugar transporter) is a transmembrane carbohydrate transporter. Stadler et al. described the first localization of a guard cell-specific Arabidopsis sugar transporter involved in carbon acquisition in these symplastically-isolated cells [39]. The timing of the transient increase in *AtSTP1* expression correlated with guard cell-specific accumulation of sucrose. Sherson et al. investigated the *in vivo* properties and function of the high-affinity monosaccharide/proton symporter AtSTP1 in Arabidopsis, showing that AtSTP1 is the major monosaccharide transporter in Arabidopsis seedlings and suggesting that active transport by AtSTP1 plays a major role at very high concentrations of exogenous sugar [40]. Our results showed that *STP1* (sugar transporter 1, GRMZM2G374812) exhibited the same expression patterns as *ZmPIP1;5* and may be involved in transmembrane carbohydrate transport. In addition, Wormit et al. showed that monosaccharide transporter1 (TMT1) is involved in vacuolar monosaccharide transport and plays a major role during stress responses [41]. Our results showed that *TMT2* (tonoplast monosaccharide transporter2, GRMZM2G083173, EntrezGene:100285573) exhibited the same expression patterns as *ZmPIP1;3*/*ZmPIP1;4* and may be involved in substrate-specific transmembrane transporter activity (GO:0022891) in developing leaves.

**Peptide**

In contrast to *ZmNIP*s, our results also showed that *OPT4* (oligopeptide transporter 4, GRMZM2G112456) exhibited the same expression patterns as *ZmPIP2;4* and may also be involved in transmembrane transport of peptides and oligopeptides. Another gene, *AtYSL2* is involved in metal-chelate transport. The investigations of Schaaf et al. support an involvement of *AtYSL2* in Fe and Zn homeostasis [42]. Our results showed that *YSL2* (yellow stripe like 2, GRMZM2G026391, EntrezGene:100273385) exhibited the same expression patterns as *ZmPIP1;6* and may be involved in the transport of peptides and oligopeptides during leaf development.

**Fatty Acid**

Signal recognition particle 54 kDa protein 3/SRP54 (SRP-54C, GRMZM2G038953) exhibited the same expression patterns as *ZmPIP2;3* and may enable the directed movement of short-chain fatty acids (less than 10 carbons) into, out of, or within cells. We also observed a significant relationship between the *ZmPIP1;1* gene and a transmembrane drug transporter (GRMZM2G079127, EntrezGene:100273132), and *ZmPIP1;3*/*ZmPIP1;4* and an auxin efflux carrier family protein (GRMZM2G050089) and a cationic amino acid transporter (GRMZM2G078292).

**Mercury**

Heavy-metal-associated domain-containing proteins (GRMZM2G155525, EntrezGene:100282516) and (GRMZM2G087101) exhibited the same expression patterns as *ZmPIP2;1* and may be involved in mercury ion transmembrane transporter activity (GO:0015097) in the developing leaf.

**Potassium**

Our results showed that the flavin-containing monooxygenase family protein/FMO family protein (GRMZM2G423886, EntrezGene:100272315) exhibited the same expression patterns as *ZmPIP2;1*, and 3-hydroxybutyryl-CoA dehydrogenase (GRMZM2G106250, EntrezGene:100191282) exhibited the same expression patterns as *ZmPIP1;3*/*ZmPIP1;4*. These may be involved in potassium ion transport (GO:0006813) in developing leaves.

**Magnesium**

Magnesium transporter CorA-like family protein (MRS2-2, GRMZM2G159295) exhibited the same expression patterns as *ZmPIP1;5* and may be involved in metal ion transmembrane transporter activity (GO:0046873) in the developing leaf.

**Sodium Ion**

*NHX2* (sodium hydrogen exchanger 2, GRMZM2G311165) exhibited the same expression patterns as *ZmPIP1;1*, *ZmPIP2;1*, *ZmTIP2;3*, *ZmTIP3;1* and *ZmTIP4;4* and may be involved in sodium ion transmembrane transporter activity (GO:0015385) during leaf development. Important evidence comes from Yokoi et al. [43], who indicated that *AtNHX2* has a major function in vacuolar compartmentalization of Na^+^.

**Zinc Ion**

MPPalpha (mitochondrial processing peptidase alpha subunit, GRMZM2G005036, EntrezGene:100280280) exhibited the same expression patterns as *ZmPIP2;1* and may be involved in catalytic/metal ion binding/metalloendopeptidase/zinc ion binding in developing leaves.

### 3.5. *ZmNIP*s and Associated Transmembrane Solute Transport

NIPs are located in intracellular membranes. ZmNIPs usually display low water permeability, in fact, these aquaporins are considered plant aquaglyceroporins [27]. We observed significant relationships between *ZmNIP* genes and the transport of solutes, including glycerol, phosphate, chloride (Cl^−^), drugs, auxin, malic acid, peptides, sugars, mercury, and metal ions such as potassium, copper, zinc, cobalt, and manganese, as shown in Figure 3.

**Glycerol**

A peroxidase 64 (GRMZM2G160327, EntrezGene:100281197) and a glycerol-3-phosphate transporter (GRMZM2G078757) exhibited the same expression patterns as *ZmNIP1;1*. Peroxidase 64 is involved in the regulation of heme binding that is dependent on intracellular accumulation of glycerol (GO:0020037). Our analysis indicate that only *ZmNIP1;1* exhibited the same expression patterns as the glycerol-3-phosphate transporter, indicating that *ZmNIP1;1* may be involved in transmembrane transport of glycerol.

**Phosphorus**

Phosphate mobilization into the plant is a complex process requiring numerous transporters for absorption and translocation of this major nutrient. In the *A. thaliana* genome, nine closely related high-affinity phosphate transporters have been identified but their specific roles remain unclear. Shin et al. showed that eight members of the Arabidopsis *Pht1* phosphate transporter family are expressed in roots, with *Pht1;1* and *Pht1;4* displaying the highest transcript levels [44]. Misson et al. demonstrated that the Arabidopsis *Pht1;4* high-affinity phosphate transporter was mainly expressed in roots growing on inorganic phosphate limiting medium [45], primarily in the epidermis. In addition, Misson et al. suggested a role for *Pht1;4* in phosphate absorption and translocation from the growth medium to the different parts of the plant. Our research showed that phosphate transporters *PHT4;2* (GRMZM2G102521) and *PHT4;6* (GRMZM2G048363, EntrezGene:100282593) exhibited the same expression patterns as *ZmNIP1;1*. At the same time, *PHT5* (GRMZM2G045473, EntrezGene:100194162) exhibited the same expression patterns as *ZmNIP2;1*. These genes may be involved in transmembrane inorganic phosphate transport in the developing leaf. Our results also showed that DEG15;endopeptidase (GRMZM2G162699, EntrezGene:100384043) and GH9A1 (hydrolyzing O-glycosyl compounds 9A1, GRMZM2G003379) exhibited the same expression patterns as *ZmNIP3:1* and may be involved in alpha-trehalose-phosphate synthase (UDP-forming) activity (GO:0003825) in developing leaves.

**Chloride**

Anion transporting proteins of the CLC type are involved in anion homeostasis in a variety of organisms. Seven chloride channel (CLC) members have been identified in the Arabidopsis genome and appear to have different roles in diverse cell organelles. Marmagne et al. have shown that AtCLC-e is targeted to the thylakoid membranes in chloroplasts, in agreement with this subcellular localization [46]. The AtCLC-f protein is localized to Golgi membranes and functionally complements the yeast *gef1* mutant, which is disrupted in the single yeast CLC gene that encodes a Golgi-associated protein [47]. CLC-b, a close relative of CLC-a, is localized to the tonoplast, and its expression is strongest in young roots, hypocotyl and cotyledons. Fecht-Bartenbach showed that *AtCLC-d* is weakly expressed in various tissues, including the root. This suggested that the luminal pH in the trans-Golgi network is adjusted by AtCLC-d-mediated transport of a counter anion such as Cl^−^ or NO_3_^−^ [48]. Our results showed that chloride channel (CLC) members CLC-f (GRMZM2G128969) and plastocyanin-like domain-containing protein (GRMZM2G139193, EntrezGene:100284694) exhibited the same expression patterns as *ZmNIP1;1*, and chloride channel protein CLC-d (GRMZM2G397836) exhibited the same expression patterns as *ZmNIP2;2*. These may be involved in chloride transmembrane transport in the developing leaf.

**Sulfate**

In addition to *ZmTIP*s and *ZmPIP*s, our results showed that *SULTR1;3* sulfate transmembrane transporter (GRMZM2G159632, EntrezGene:541917) and *SULTR3;1* (GRMZM2G158013, EntrezGene:100382058) exhibited the same expression patterns as *ZmNIP2;2* and may be involved in sulfate transmembrane transport in developing leaves.

**Auxin**

Lewis et al. have investigated ABCB19 from *A. thaliana*, which belongs to the B group of the ATP-binding cassette (ABC) transporter superfamily. ABCB19 mediates polar auxin transport in stems and roots, and cotyledon expansion during the establishment of photoautotrophic growth depends on ABCB19-mediated auxin import [32]. Our results showed that ABCB19:ATPase multidrug resistance protein 11 (GRMZM2G072850) exhibited the same expression patterns as *ZmNIP1;1* and may be involved in ABC transporter and ATPase activity coupled to transmembrane movement of substances (GO:0042626), and the G-protein coupled receptor protein signaling pathway (GO:0007186) in the developing leaf. Our results also showed that OsSAUR12:auxin-responsive SAUR gene family member (GRMZM2G154332, EntrezGene:100284645) exhibited the same expression patterns as *ZmNIP2;2*.

**Peptide**

In Arabidopsis, *AtPTR2*, a peptide transport gene, has been suggested to have an important physiological role in plant growth and development [49]. After evaluating the function of this transporter, Song et al. suggested that AtPTR2-B may play a general role in plant nutrition [50]. Our results showed that peptide transporter PTR2-B (GRMZM2G378604, EntrezGene:100281589) and plastocyanin-like domain-containing protein (GRMZM2G139193, EntrezGene:100284694) exhibited the same expression patterns as *ZmNIP1;1* and may be involved in oligopeptide transport (GO:0006857) in developing leaves. In addition, our results also showed that OPT7 (oligopeptide transporter 7, GRMZM2G479703, EntrezGene:100383021) exhibited the same expression patterns as *ZmNIP2;1*. There is evidence that AtOPTs mediate the uptake of tetra- and pentapeptides [51].

**Sugar**

A wide range of sugars including hexoses, pentoses, tetroses, a sugar acid, and sugar alcohols, but not disaccharides, induced inward currents in oocytes expressing *AtPLT5*. Reinders et al. showed that *AtPLT5* encodes an ion-coupled uptake transporter [52]. Our results showed that *ATPLT5* (polyol transporter 5, GRMZM2G153920, EntrezGene:100281055) exhibited the same expression patterns as *ZmNIP2;1* and may be involved in sugar and substrate-specific transmembrane transporter activity (GO:0022891).

From our results, we also observed significant relationships between the *ZmNIP1;1* gene and a transmembrane drug transporter (GRMZM2G069098, EntrezGene:100286164), and *ZmNIP2;1* and other drug transmembrane transporters (GRMZM2G043075, GRMZM2G115105). These may be involved in transmembrane drug transport (GO:0006855). Malic acid transport protein SLAH3 (GRMZM2G061469, EntrezGene:100281575) also had a significant relationship with *ZmNIP1;1*.

**Potassium**

Potassium (K^+^) is a major plant nutrient required for growth and development. K^+^ uptake in the high-affinity range of concentrations and its components have been widely studied. In *A. thaliana*, the AtHAK5 transporter and the AtAKT1 channel have been shown to be the main transport proteins involved in this process [53]. Growth analysis shows that AtHAK5 plays a role during severe K^+^ deprivation. Under K^+^-deficient conditions, in the presence of Cs^+^, the high-affinity K^+^ transporter AtHAK5 and the inward-rectifier K^+^ channel AtAKT1 have been attributed to K^+^ uptake in Arabidopsis [54]. Our results showed that *HAK5* (high potassium transporter 5, GRMZM2G455817) exhibited the same expression patterns as *ZmNIP2;1* and may be involved in potassium ion transmembrane transporter activity (GO:0015079) in the developing leaf. Our results also showed that *PDS3* (phytoene desaturase 3, GRMZM2G088601) exhibited the same expression patterns as *ZmNIP2;1* and may be involved in potassium ion transport (GO:0006813) during leaf development. Potassium transporter 8, KT2 (GRMZM2G125387) exhibited the same expression patterns as *ZmNIP1;1* and may also be involved in potassium ion transmembrane transporter activity (GO:0015079).

**Calcium**

The magnitude and duration of cytosolic Ca^2+^ release can potentially be altered by changing the rate of Ca^2+^ efflux. In plant cells, Ca^2+^ efflux from the cytoplasm is mediated by H^+^/Ca^2+^-antiporters and two types of Ca^2+^-ATPases. ACA2 was recently identified as a calmodulin-regulated Ca^2+^-pump located in the endoplasmic reticulum [55]. In our results we observed significant relationships between the *ZmNIP2;2* gene and calcium ion transmembrane transporters ACA4 (GRMZM2G104730) and ACA2(GRMZM2G352695). These may be involved in ATPase activity coupled to transmembrane ion movement phosphorylative mechanisms (GO:0015662) and transmembrane calcium ion transporter activity (GO:0015085).

We also observed significant relationships between *ZmNIP* genes and metal ion transporters. *ZmNIP1;1* showed a significant relationship with a copper ion transmembrane transporter (GRMZM2G139193, EntrezGene:100284694), which is a plastocyanin-like domain-containing protein that may also be involved in copper ion binding (GO:0005507). *ZmNIP1;1* showed a significant relationship with a mercury ion transporter (GRMZM2G150450, EntrezGene:100281799) and may be involved in mercury ion transmembrane transporter activity (GO:0015097). *ZmNIP2;3* showed a significant relationship with ACBP6 (acyl-CoA-binding protein 6, GRMZM2G344634, EntrezGene:100281027) and may be involved in cadmium transmembrane transport. ROP9 (rho-related protein 9, AC209819.3_FG012, EntrezGene:542503) exhibited the same expression patterns as *ZmNIP1;1* and may be involved in cobalt ion homeostasis (GO:0006877). This GO category also includes zinc ion transporters and divalent metal ion transporters involved in manganese homeostasis.

## 4. Discussion

In the present study, the results show the expression profiles of aquaporins along a leaf developmental gradient were mostly statistically significant, and expression differed by orders of magnitude. *ZmTIP4;3, ZmTIP4;1, ZmTIP1;1, ZmSIP1;1, ZmSIP1;2, ZmSIP2;1, ZmPIP2;7, ZmNIP5-1* showed hardly any or no expression in four developmental maize leaf zones. *ZmTIP4;4, ZmTIP3;2, ZmNIP1;1, and ZmNIP3;1* were specifically expressed in the mature leaf zone, while ZmNIP2;1 and ZmTIP4;2 were specifically expressed in the leaf basal. Based on the gene expression profiles of the 24 maize aquaporins along a leaf developmental gradient, a coexpression network-based approach was used to construct coexpression networks. The coexpression networks are partitioned into two modules, indicating a strong modular structure. one modules includes *ZmPIP2;1, ZmNIP3;1, ZmPIP1;6, ZmPIP2;4, ZmPIP1;3/ZmPIP1;4, ZmTIP3;2, ZmPIP2;3, ZmPIP1;1, ZmPIP2;2, ZmNIP1;1, ZmTIP2;3, ZmTIP1;2, ZmTIP3;1, ZmTIP4;4,* which showed the highest expression in the basal and transitional zone showed much lower or hardly detectable expression in the maturing and mature zone. While the opposite applied to another modules which are *ZmPIP1;5, ZmPIP2;5, ZmNIP2;3, ZmNIP2;1, ZmTIP4;2, ZmTIP2;1, ZmTIP2;2, ZmPIP2;6, ZmNIP2;2,* those genes showed the highest expression in maturing and mature leaf tissue. These results do not seem surprising considering that there are two groups of aquaporins, one positively and the other negatively regulated during different stages of leaf development.

After examining the dynamic expression patterns of aquaporins in four maize leaf developmental zones, we were interested in studying the correlations of aquaporins and nutrient transporters along a leaf developmental gradient. The results described the correlations of ZmTIPs, ZmPIPs, ZmNIPs, and associated transmembrane solute transport in Section 3 provided novel insights and showed a significant specificity correlation between aquaporins and transmembrane solute transporters in the developing maize leaf.

### 4.1. Specific Maize Aquaporins Are Related to Specific Transmembrane Transporter Families in Developing Leaves

**Figure 4:**
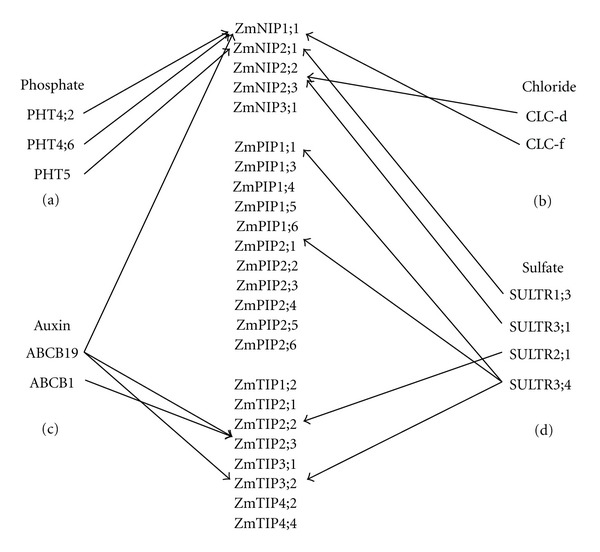
Specific aquaporins are related to a specific transmembrane solutes(ions) transporters in developing maize leaf (a) phosphate transporter (b) chloride channel members with maize aquaporins (c) ABC transporters and multidrug resistance protein (d) sulfate transmembrane transport with maize aquaporins.

It is proposed that during evolution, plant aquaporins diversified in terms of both the specificity of their expression in plant tissues and their water permeation properties, while maintaining their ability to be induced by the activities of their transmembrane solute transporters. Some aquaporins are more related to specific developmental stages and/or organs. In this study, we confirmed that sulfate transmembrane transporters show specific correlation to certain maize aquaporins, as shown in Figure 4. SULTR1;3 shows a significant relationship with *ZmNIP2;1*, SULTR3;1 with *ZmNIP2;2*, SULTR2;1 with *ZmTIP2;2*, and SULTR3;4 with *ZmTIP3;2*, *ZmPIP1;1* and *ZmPIP2;1*. This suggests that these maize aquaporins are related to a specific sulfate transmembrane transporter family, SULTR, in the developing leaf.

With chloride channel (CLC) members, our results show that not only does CLC-f exhibit the same expression patterns as *ZmNIP1;1* in developing leaves, but also chloride channel protein CLC-d, which is believed to be involved in inorganic phosphate transmembrane transport, displays the same expression patterns as *ZmNIP2;2*.

Our analysis also reveals that phosphate transporters *PHT4;2* and *PHT4;6* exhibit the same expression patterns as *ZmNIP1;1*, and *PHT5* the same as *ZmNIP2;1*, suggesting that these genes may be involved in inorganic phosphate transmembrane transport during leaf development.

### 4.2. Overlapping Patterns of Expression for Some of Transmembrane Solute Transport Family Members

Overlapping patterns of expression for some of the transmembrane solute transport family members were evident in our analysis. Lavin-containing monooxygenase family protein/FMO family protein not only exhibits the same expression patterns as *ZmTIP2;3*, but also *ZmPIP2;1*, suggesting it may be involved in potassium ion transport (GO:0006813) in the developing leaf. ABCB19:ATPase multidrug resistance protein 11 shows the same expression patterns as *ZmNIP1;1*, *ZmTIP2;3,* and *ZmTIP3;2*, suggesting that these aquaporins may be involved in ABC-mediated transport and multidrug resistance systems. Peptide transporter PTR2-B proton-dependent oligopeptide transport (POT) family protein exhibits the same expression patterns as *ZmTIP2;3* and *ZmNIP1;1*, suggesting that these genes may be involved in oligopeptide transport (GO:0006857) during leaf development. ATPLT5 (polyol transporter 5) exhibits the same expression patterns as *ZmNIP2;1* and *ZmTIP3;2*, suggesting they may be involved in the transport of sugars.

Accurate prediction in our approach depends on the conditions associated with each gene. To evaluate the predictive capacity, we examined previously published research work. Important evidence about the reliability of our systems level analysis comes from Kataoka et al. [30]. Sulfate transmembrane transporters include a family of high-affinity sulfate transporters, with members *SULTR1;1*, *SULTR1;3*, *SULTR1;2*, *SULTR3;1*, *SULTR3;2*, *SULTR3;4*, *SULTR3;5*, *SULTR4;1,* and *SULTR4;2*. Kataoka et al. showed that in Arabidopsis, SULTR3;5 was colocalized with the SULTR2;1 low-affinity sulfate transporter in xylem parenchyma and pericycle cells in roots. The root-to-shoot transport of sulfate is restricted in *sultr3;5* mutants, and under conditions of high *SULTR2;1* expression in the roots after sulfur limitation, coexpression of *SULTR3;5* and *SULTR2;1* provides maximal sulfate transport activity. This facilitates retrieval of apoplastic sulfate from the xylem parenchyma cells into the vasculature of Arabidopsis roots and may contribute to the root-to-shoot transport of sulfate. In this study, we found that *SULTR3;1* (sulfate transporter 3;1, GRMZM2G158013, EntrezGene:100382058) exhibits the same expression patterns as *ZmNIP2;2*, and *SULTR2;1* (sulfate transporter2;1, GRMZM2G042171) the same as *ZmTIP2;2*. indicating that these genes are coexpressed and involved in transmembrane sulfate transport in the developing leaf. Other evidence comes from recent investigations on how the structural features of plant aquaporins at atomic resolution influence their substrate selectivity. Our results are in accord with data reported by [6] and [8].

## 5. Conclusion

The mechanisms controlling the selectivity of aquaporins and nutrient transporters in most plants have been widely studied [6, 8]. However, these studies have focused on aquaporin structures at atomic resolution. Advances in postgenomic technologies and their use by the scientific community are generating increasing quantities of high quality genome-wide transcriptomic data sets. Deposition of these data sets into publicly accessible online databases enables researchers to analyze the collated data and uncover novel information. Therefore, there is a great need for additional analytical approaches to maximize the return on the large collective investment made in data generation. Here, we present a genome-wide analysis and coexpression network-based approach as a powerful novel associative tool for the investigation and prediction of gene function using gene expression data from developing maize leaves. The analysis demonstrates a systems-level correlation between aquaporins, nutrient transporters, and the homeostasis of mineral nutrients in the developing maize leaf. This computational methodology represents a useful alternative approach for the extraction of biological knowledge from existing data. Our results also provide a resource for further studies into the physiological function of these aquaporins.

## Supplementary Material

Supplemental file1: 812 maize transmembrane transport genes and expression along the leaf developmental gradient.
Supplemental file2: 33 identified aquaporins in maize from the MaizeGDB and Affymetrix GeneChip Maize Genome Array.
Supplemental file3: Simiar pattern of maize aquaporins and transmembrane solutes(ions) transporters in developing maize leaf.

## Figures and Tables

**Table 1:** Dynamic expression patterns of aquaporins in four developmental maize leaf zones.

Aquaporins family	kgeneID	Accession no.	uid	NCBI-GeneID	Expression patterns of maize aquaporins			
					along a leaf developmental gradient (RPKM^+^)			
					Basal	Transitional	Maturing	Mature
ZmNIP1;1	GRMZM2G041980	AAK26750	5	zma:542741	284.4251	55.78293	1.32962	0.604141
ZmNIP2;1	GRMZM2G028325	AAK26751	5	zma:542643	0.738541	3.736517	108.7187	151.1207
ZmNIP2;2	GRMZM2G137108	AAK26752	6	zma:541884	12.83963	65.19496	110.8307	191.0704
ZmNIP2;3	GRMZM2G081239	AAK26849	9	zma:542497	1.390615	38.11073	19.33411	28.52731
ZmNIP3;1	GRMZM2G176209	AAK26753	1	zma:541885	10.89309	0.622895	0.379714	0.028008
ZmPIP1;1	GRMZM2G174807	Q41870	2	zma:542434	1373.24	624.1622	551.3903	696.9544
ZmPIP1;3	GRMZM2G392975	AAK26754	4	zma:541886	320.3079	208.3987	120.4039	143.314
ZmPIP1;4	GRMZM2G392975	AAK26754	4	zma:541886	320.3079	208.3987	120.4039	143.314
ZmPIP1;5	GRMZM2G081843	AAK26756	4	zma:542014	6.403676	129.4154	92.4027	203.5336
ZmPIP1;6	GRMZM2G136032	AAK26757	9	zma:541887	8.864766	2.943776	5.884045	10.33727
ZmPIP2;1	GRMZM2G014914	AAK26758	7	zma:541888	863.1962	222.715	133.0945	270.8236
ZmPIP2;2	GRMZM2G092125	AAK26759	2	zma:542644	365.4722	72.14911	103.3769	246.2429
ZmPIP2;3	GRMZM2G081192	AAK26760	4	zma:541889	99.41061	97.24697	32.23512	64.3703
ZmPIP2;4	GRMZM2G154628	AAK26761	5	zma:541890	159.1274	254.0077	62.26854	66.46106
ZmPIP2;5	GRMZM2G178693	AAD28761	2	zma:542619	34.77607	26.43263	61.35096	248.551
ZmPIP2;6	GRMZM2G047368	AAK26762	7	zma:541891	28.2033	18.13528	53.17567	199.4343
ZmTIP1;2	GRMZM2G168439	AAK26767	8	zma:541893	124.288	11.90748	13.80556	12.93096
ZmTIP2;1	GRMZM2G027098	AAK26768	4	zma:541894	84.54834	136.8424	174.5521	380.7838
ZmTIP2;2	GRMZM2G056908	AAK26769	5	zma:541895	4.553362	5.91993	7.847642	21.60773
ZmTIP2;3	GRMZM2G125023	AAK26770	2	zma:541687	16.41429	1.501289	0.66028	0.912982
ZmTIP3;1	GRMZM2G305446	AAK26771	5	zma:541896	1.72853	0.06663	0*	0*
ZmTIP3;2	GRMZM2G103983	AAK26848	1	zma:541912	15.56036	0.91543	0.686821	1.139873
ZmTIP4;2	GRMZM2G108273	AAK26773	8	zma:541898	0.028412	0.11653	8.459535	13.80056
ZmTIP4;4	GRMZM2G093090	AAK26775	3	zma:542647	10.02319	0.695458	0.034258	0*

Basal (1 cm above the leaf three ligule), transitional (1 cm below the leaf two ligule).

Maturing (4 cm above the leaf two ligule), mature (1 cm below the leaf three tip).

^+^RPKM: Reads per kilobase of exon model per million mapped reads.

*Those genes showed hardly any or no expression in leaf tissue.
